# Use of Lean Response to Improve Pandemic Influenza Surge in Public Health Laboratories

**DOI:** 10.3201/eid1801.101485

**Published:** 2012-01

**Authors:** Judith L. Isaac-Renton, Yin Chang, Natalie Prystajecky, Martin Petric, Annie Mak, Brendan Abbott, Benjamin Paris, K.C. Decker, Lauren Pittenger, Steven Guercio, Jeff Stott, Joseph D. Miller

**Affiliations:** Provincial Health Services Authority, Vancouver, British Columbia, Canada (J.L. Isaac-Renton, Y. Chang, M. Petric, A. Mak, B. Abbott, J. Stott);; University of British Columbia, Vancouver (J.L. Isaac-Renton, N. Prystajecky, M. Petric);; Booz Allen Hamilton, McLean, Virginia, USA (B. Paris, K.C. Decker, L. Pittenger);; Public Health Agency of Canada, Winnipeg, Manitoba, Canada (S. Guercio);; Centers for Disease Control and Prevention, Atlanta, Georgia, USA (J.D. Miller)

**Keywords:** Pandemic, influenza, viruses, Lean response, public health laboratory, surge capacity, modeling, pandemic (H1N1) 2009

## Abstract

A novel influenza A (H1N1) virus detected in April 2009 rapidly spread around the world. North American provincial and state laboratories have well-defined roles and responsibilities, including providing accurate, timely test results for patients and information for regional public health and other decision makers. We used the multidisciplinary response and rapid implementation of process changes based on Lean methods at the provincial public health laboratory in British Columbia, Canada, to improve laboratory surge capacity in the 2009 influenza pandemic. Observed and computer simulating evaluation results from rapid processes changes showed that use of Lean tools successfully expanded surge capacity, which enabled response to the 10-fold increase in testing demands.

## Abstract 2

These tools enabled laboratory response to the 10-fold increase in testing demands.

A novel influenza A (H1N1) virus was detected in Mexico and the southwestern United States in early April 2009 (*1*). Within days after confirmation that this virus was circulating in the western Canadian province of British Columbia, the number of requests for influenza diagnostic tests rapidly increased. It became evident that current operations would not enable the British Columbia Public Health Microbiology & Reference Laboratory (PHMRL), the major provider of influenza diagnosis for this province, to meet testing demands. We describe Lean processes that were implemented to rapidly expand surge capacity.

## Methods

### Prepandemic Testing for Influenza

The PHMRL serves the entire health care system for the western Canadian province of British Columbia (population 4.45 million). Pandemic planning lead by the Canadian Public Health Laboratory Network included implementation of a reverse transcription PCR (RT-PCR) platform. Before the pandemic, sample data were entered into the Laboratory Information System (LIS) and bar-coded in the Central Processing & Receiving section; the accessioned respiratory samples were then transported to the Virology Laboratory, located 3 floors away. In the Virology Laboratory, 1 laboratory assistant organized the samples and transferred aliquots into labeled tubes.

Testing was conducted by 1 medical laboratory technologist; tasks included nucleic acid extraction, RT-PCR, analysis of results, and report of results into the LIS. One easyMag extractor (bioMérieux, Marcy l’Etoile, France) (capacity 22 patient samples) and 1 ABI 7900 RT-PCR machine (Applied Biosystems, Foster City, CA, USA) (capacity 92 patient samples) were used. These processes were conducted 10.5 h/d, 6 d/wk during the normal British Columbia influenza season (September–March) by 1 laboratory assistant and 2 medical laboratory technologists (1 technologist on each of 2 shifts). These assignments enabled PHMRL to meet prepandemic demand for influenza testing. Test results were available on the same day as arrival in PHMRL, except for weekends. Volumes seldom exceeded 50 samples/d.

### Initial Pandemic Response

Once the pandemic was confirmed, the need for an RT-PCR to reliably detect the novel subtype was the highest priority. Because of Canadian Public Health Laboratory Network leadership (*2*), followed by application of the recommendations by the provincial public health laboratory, an RT-PCR for influenza targeting the conserved matrix gene had been used in our Virology Laboratory for several years. However, it was able to identify only the new virus as influenza A; the subtyping RT-PCR (for seasonal subtype H1N1 virus) was not able to identify the pandemic virus. Using genomic information provided in GenBank by the Centers for Disease Control and Prevention (Atlanta, GA, USA), we addressed this priority by sequencing the M gene amplicons generated by the existing RT-PCR (*3*), which enabled initial testing response. Further improved assays were introduced later and validated against an RT-PCR provided by Canada’s National Microbiology Laboratory. Commercially available point-of-care devices were not recommended in Canada except for use in defined small and remote communities.

### Response to Surge by Using Lean Methods

With an assay that could detect the novel pathogen, expansion of the overall surge capacity of the PHMRL was the critical next step. Medical and operations directors met with a multidisciplinary team of scientific and senior technical staff to determine how to address this challenge. Although additional equipment and reagents were immediately approved for purchase, hiring of additional staff was not. Improving efficiencies by changing workflow processes was necessary. Before the onset of the pandemic, PHMRL staff had been trained in Lean methods (*4**,**5*), and many had participated in activities to enhance laboratory processes. Lean principles have been used extensively in manufacturing industries and more recently applied to various health care domains, including facility design (*6*) and redesign (*7**,**8*), patient flow (*9*), infection control (*10*), and clinical pathology laboratories (*11**–**13*). A key principle of Lean thinking is that work can be done more efficiently by identifying and eliminating waste to optimize the workflow. Lean also focuses on meeting client needs and involving support staff in making ongoing improvements to their work processes.

**Figure 1:**
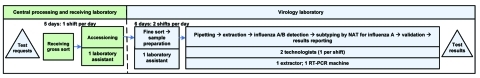
Seasonal influenza testing processes (pre-kaizen value stream). NAT, nucleic acid amplification techniques; RT-PCR, reverse transcription PCR.

A Lean Team comprising medical, scientific, senior, and bench-level technical staff and some administrative and support personnel was set up to apply Lean methods (Table) to the influenza laboratory workflow. The team first created a flowchart of baseline laboratory tasks to assess processes, staff assignments, and sample batch sizes. This visually displayed value stream (Table) included all discrete steps in the laboratory workflow (Figure 1) for influenza detection and subtyping with the resources and time required for each step. Process information from the separate traditional laboratory work areas (Central Processing & Receiving on the ground floor and the Virology Laboratory on the fourth floor) were included. Review showed that most waste occurred between each discrete process step (wait times, setup time) as the 1 medical laboratory technologist in the Virology Laboratory took each batch of samples through the steps of extraction, RT-PCR, and reporting. The addition of 2 ABI 7900 RT-PCR analyzers and a high-throughput nucleic acid ABI MagMAX extractor (Applied Biosystems) with capacity of 92 patient samples highlighted current workflow process inefficiencies.

**Table:** Lean methods used to improve laboratory workflow

Method	Definition
Kaizen	The incremental, ongoing improvement process aimed at creating efficiencies and improving all functions. Productivity increases are attributed to eliminating waste in the system and having all staff participate in the process.
Value stream mapping	A technique used to identify the materials and information required in each step of a process to deliver a product to a consumer
Andon	A visual cue used to monitor functions and to notify workers of a quality or process issue
5S	The process of sorting, storing, sweeping, sustaining, and standardizing, which results in a visually managed environment that allows staff to perform tasks more efficiently

**Figure 2:**
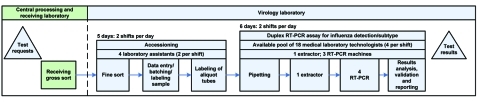
Pandemic (H1N1) 2009 testing processes (post-kaizen with the flow cells depicted as processes occurring within a box). RT-PCR, reverse transcription PCR.

A kaizen session (Table) working with staff in the Virology Laboratory originated the concept of flow cells in which, instead of all steps in the influenza detection process being performed by 1 medical laboratory technologist, each task in the work flow was separated into individual flow cells. Separate staff members were dedicated to each cell. Within the new process (Figure 2), the new visually linked work units created more repetitive, standardized procedures. Some of the flow cells with shorter cycle times (such as data entry) could then be performed more times per day by the new flow cell teams. Their outputs were balanced with other flow cells with longer cycle times (such as RT-PCR). Flow cells, each with a new standard operating procedure and training checklist, were implemented with the support of laboratory staff and included a data entry cell; a labeling cell; a pipetting/aliquoting cell; and extraction, RT-PCR, and reporting cells. Put in place in the appropriate value stream sequence within the Virology Laboratory, they formed a new rapidly scalable and standardized work process.

The Lean Team also implemented changes in staffing to match improvements in workflow processes so that laboratory assistants were assigned the tasks of sample receiving, sorting, and triaging; data entry (accessioning into the LIS); and other support duties, and medical laboratory technologists were assigned to sample preparation, DNA extraction, RT-PCR, result validation and result reporting. Samples were batched into groups of 92 to optimize the plate platform.

Other efficiencies identified by the Lean Team included redeploying laboratory assistants, who were responsible for respiratory sample triage and data entry but worked 3 floors away, to work in the Virology Laboratory. This change also ensured that questions related to samples could be immediately addressed. Three additional laboratory assistants now joined the existing Virology Laboratory assistant in 2 shifts (2 laboratory assistants per shift). To accommodate the new workflow and the unprecedented testing demand, medical laboratory technologists from other laboratories in the PHMRL were cross-trained. Reducing the influenza detection process into discrete flow cell units meant that these additional medical laboratory technologists could quickly be trained and gain documented competency in the procedures within each flow cell. The new simplified, specific task focused workflow now enabled 8 medical laboratory technologists in 2 shifts (4 medical laboratory technologists per shift in 1 flow cell each) that could draw from a developing reserve pool of cross-trained staff.

Applying another Lean principle, the 5S approach (Table), we reorganized and clearly labeled bench tops and other work areas to demarcate discrete workflow steps. This change enabled visual management of the samples now being processed in a single site (Virology Laboratory).

### Evaluation by Modeling Analysis

As part of a national laboratory initiative, PHMRL collaborated with the consulting firm Booz Allen Hamilton (McLean, VA, USA) to assess pandemic surge capacity. Model inputs, including information equipment, sample volumes, tests, work shifts, task times, and staff number and type, were collected, and prepandemic influenza data from the previous year’s peak month (January 2008) were compared with peak pandemic month (October 2009). Data were used in a discrete event model in the ExtendSim Process Modeling Environment (Imagine That, Inc., San Jose, CA, USA), a customized process model replicating the PHMRL pandemic processes. FluLabSurge (*14*), an Excel-based software program (Microsoft, Redmond, WA, USA), was also used to provide a theoretical estimate of test volumes from a 1968-level pandemic (moderate severity) when corrected for current British Columbia population. Other model assumptions were 1) staff absenteeism was constant at 5%; 2) staff were considered fully used at 85% (accounting for breaks, administrative duties, and consultations with co-workers); 3) the simulation was run for a 180-day pandemic; and 4) reagents were not considered a limiting factor.

## Results

**Figure 3:**
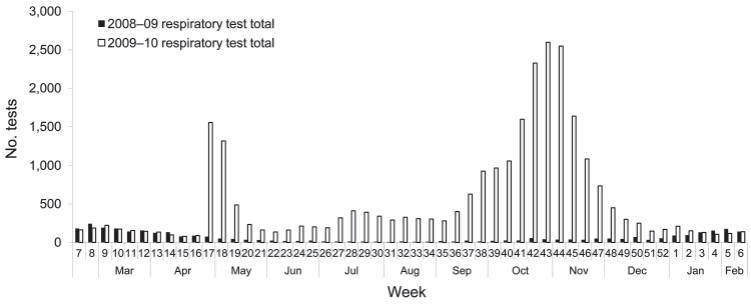
Number of respiratory samples tested by the British Columbia (Canada) Public Health Microbiology & Reference Laboratory in the 2008–09 influenza season compared with the pandemic (H1N1) 2009.

The new Lean-based flow cell configuration with additional equipment, 3 additional laboratory assistants, 3 additional medical laboratory technologists per shift (by using newly cross-trained staff from other PHMRL laboratories), and an extended workday (13.5 h), enabled large numbers of samples to be tested each day. Implementation of the new flow cells for staffing balanced the additional equipment capacity, creating a smooth workflow of repetitive tasks that resulted in decreased sample waiting times and more efficient throughput. This rapid response, implemented within a few days after the initial Lean team meeting, enabled the laboratory to meet the surge in demand; the number of tests and percentage of samples positive for influenza during the pandemic were beyond anything the laboratory had ever experienced (Figure 3). Compared with a maximum daily test volume of 53 in the 2008 routine influenza season, the corresponding maximum in the 2009 pandemic was 573 (November 3, in the second wave).

**Figure 4:**
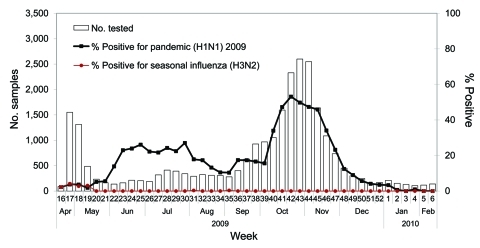
Simulated pandemic FluSurge level of daily test volume demand showing British Columbia (Canada) Public Health Microbiology & Reference Laboratory seasonal capacity estimated to be 231 samples per day and postemergency (kaizen) pandemic capacity 528 samples per day.

**Figure 5:**
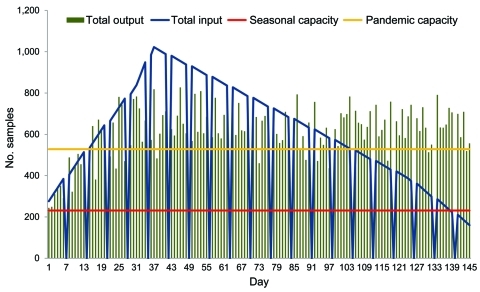
Simulated back-log using seasonal and postemergency (kaizen) pandemic processes with a FluLabSurge (1968 pandemic) level of expected test demand for population, British Columbia, Canada.

Results of modeling the surge that used the 2 different operational processes (prepandemic and pandemic modes) showed that an average of 231 samples per day theoretically could have been managed (with same-day testing) by using traditional seasonal influenza processes and resources. Although the actual maximum sample volume was only 53 samples per day in our experience, modeling was thus able to quantitate the potential capacity by using standard prepandemic resources and processes. Modeling again was able to quantitate the capacity increase after improvements that used Lean methods, calculating the testing capacity to be 528 samples per day (Figure 4) based on flow cell processes. Modeling also showed that, had the laboratory actually been faced with a 1968-level pandemic with its much higher sample surge, demand would have far exceeded output throughout the pandemic wave by using seasonal resources (Figure 5).

Daily reports based on another Lean concept, andon (Table), relayed the pandemic status to management. Information on test volumes; influenza virus subtypes; positivity rates; turnaround times; and status of equipment, reagents, and staffing were made available. Successful implementation of process improvements by using Lean methods rely heavily on acceptance and uptake by staff; after an initial discussion and decision to apply Lean principles, kaizen sessions were held with staff on the work floor. Other internal communications included staff training (and technical competency assessment) sessions, staff meetings, acknowledgment of contributions on bulletin boards, and regular internal newsletters. All PHMRL staff were invested in the pandemic because contributions came from all laboratory sections to support the Virology Laboratory.

## Discussion

Although medical laboratories, including public health laboratories, are critical to the health care system, their role is often not fully recognized. In addition to affecting >80% of clinical decisions (*15*), laboratories provide vital functions related to community-level health care. The 2009 pandemic underscores the contributions of laboratories and the need for continuous improvements by using methods such as Lean. Such preparedness provides staff with the ability to make rapid improvements, such as in surge capacity, and to share innovations and improvements (*16*). Although the initial response was development and implementation of the new RT-PCR by scientific staff and although prior preparation as a network was fundamental to the pandemic response, training local laboratory staff in Lean methods enabled these tools to be applied for rapid improvements in the PHMRL influenza detection process. In particular, implementation of flow cells to standardize repetitive tasks and to balance the additional equipment acquired resulted in meeting acceptable turnaround times despite the unprecedented influx of samples. Results of the rapid response were shown by computer process modeling to have been critical. Without making the changes noted above, the PHMRL would have been unable to meet the testing demands in the 2009 pandemic.

As fiscal constraints in health care continue to grow, the system needs to recognize the contributions—and the needs—of the complex laboratory system. We demonstrated how the application of Lean tools can rapidly improve processes required for surge capacity. They can be used between events for finding ongoing required system efficiencies. Computer process modeling that confirmed the Lean Team’s work also appears to be a tool that, with further refinements, could be used to predict ways of improving other laboratory processes and to guide further change.
